# Gabapentin as a Symptomatic Modifier in Median Arcuate Ligament Syndrome: A Case Report and Assessment of Treatment Modalities

**DOI:** 10.7759/cureus.26401

**Published:** 2022-06-28

**Authors:** Abdurrahman F Kharbat, Ranger Kile, Alfred Kankam, Bernardo Galvan, Katherine G Holder, Basem Soliman

**Affiliations:** 1 Department of Surgery, Texas Tech University Health Sciences Center, Amarillo, USA

**Keywords:** dunbar syndrome, neurogenic, atypical mals, gabapentin, median arcuate ligament syndrome

## Abstract

Median arcuate ligament syndrome (MALS) is a rare constellation of neurogenic gastrointestinal (GI) symptoms resulting from compression of the celiac trunk and celiac plexus by the median arcuate ligament. MALS is characterized by nonspecific symptoms including nausea, vomiting, diarrhea, bloating, unintentional weight loss due to food aversion, and postprandial epigastric abdominal pain. We present a case of atypical, chronic MALS that confounded clinicians for over a decade and led to various misdiagnoses, including early-onset Parkinson’s disease. Of the constellation of symptoms that MALS may present with, postprandial epigastric pain is a classic symptom and increases the index of suspicion for the diagnosis; however, the absence of the classic symptom of postprandial epigastric pain and the predomination of nonspecific GI symptoms and syncope in our patient further clouded clinicians’ ability to diagnose MALS. Upon further investigation, we elucidated a link between gabapentin, which our patient was chronically prescribed, and its efficacy in decreasing neurogenic hypersensitivity in the GI tract. Our case and the implications of gabapentin use to decrease neurogenic pain from MALS represents a novel addition to the literature on MALS treatment modalities and elucidates new avenues for continued research in the use of gabapentin as a symptom-modifying agent in the nonoperative and preoperative treatment of MALS.

## Introduction

Median arcuate ligament syndrome (MALS), also known as celiac artery compression syndrome or Dunbar syndrome, is a rare constellation of gastrointestinal (GI) symptoms resulting from extrinsic compression of the celiac trunk and plexus/ganglion by the median arcuate ligament and diaphragmatic crura. Most seen in 30- to 50-year-old women with thin body habitus, MALS typically presents with varying degrees of nonspecific symptoms including nausea, vomiting, diarrhea, bloating, unintentional weight loss due to food aversion, and, most notably, postprandial abdominal pain (primarily epigastric) [1-3]. Given the nonspecific symptoms and resemblance to other more common abdominal disorders, the diagnosis of MALS is challenging and generally considered to be one of exclusion.

The median arcuate ligament is a fibrous band connecting the diaphragmatic crura anteriorly, forming the aortic hiatus superior to the celiac trunk. In the presence of an unusually superior origin of the celiac trunk and/or unusually inferior insertion of the diaphragmatic crura, the median arcuate ligament may subsequently overlap and compress the celiac trunk and plexus/ganglion [4,5]. Although first described anatomically in 1917 by Lipshutz, the pathophysiology of MALS symptoms remains unknown [6]. Classic theory suggests that epigastric pain results from foregut ischemia caused by compression of the celiac artery. However, it has also been suggested that vascular steal syndrome following celiac artery compression may lead to midgut ischemia [7,8]. In contrast, others argue that the pain is neuropathic in origin and results from chronic compression and subsequent overstimulation of the celiac plexus/ganglion. This leads to irritation of the sympathetic visceral pain fibers and/or chronic splanchnic vasoconstriction and ischemia [9]. Although classically considered a vascular disorder, MALS is now often viewed as primarily a neurogenic condition [8].

Owing to its rarity, nonspecific presentation, and unknown pathogenesis, best practices for the diagnosis and intervention of MALS have yet to be defined in the literature. Current evidence suggests that duplex abdominal ultrasound during inspiration and deep expiration should be used as an initial assessment for compression of the celiac trunk [10-12]. Additional studies that may be useful in diagnosis include computed tomography (CT) angiography and magnetic resonance angiography [4,13]. Once diagnosed, percutaneous celiac plexus/ganglion block should be used to simulate decompression and determine potential surgical candidates [2,14]. Those who do not experience relief following neural blockade have a lower likelihood of success with a surgical intervention [8]. First described by Harjola et al. in 1963, the open release of the median arcuate ligament with removal and neurolysis of the celiac plexus/ganglion is the conventional approach to surgical intervention for MALS [15,16]. In recent years, however, laparoscopic decompression and neurolysis have become the accepted standard of care [13]. Not surprisingly, robotic-assisted release and decompression with neurolysis are now showing excellent promise given the ability for enhanced microdissection at the base of the celiac trunk [17,18]. Even with current advancements in diagnostic and management techniques, surgical intervention is not always clinically successful. Nonetheless, it has been reported that 75% to 85% of patients experience resolution of symptoms following surgery [19].

## Case presentation

A 33-year-old male presented to the vascular surgery clinic with a 13-year history of intermittent syncopal episodes associated with nausea, vomiting, and diffuse/poorly localized abdominal pain that was previously thought to be an irritable bowel syndrome. He reported that the episodes occurred intermittently, were not specifically associated with meals, and were accompanied by diffuse body aches. His medical history included a diagnosis of attention-deficit hyperactivity disorder (ADHD) since childhood and restless leg syndrome secondary to traumatic compartment syndrome and subsequent fasciotomy of the left lower extremity seven years prior. He also endorsed left lower extremity neuropathic pain controlled with gabapentin. His family history was significant for epilepsy in his mother. His remaining histories were unremarkable.

**Figure 1:**
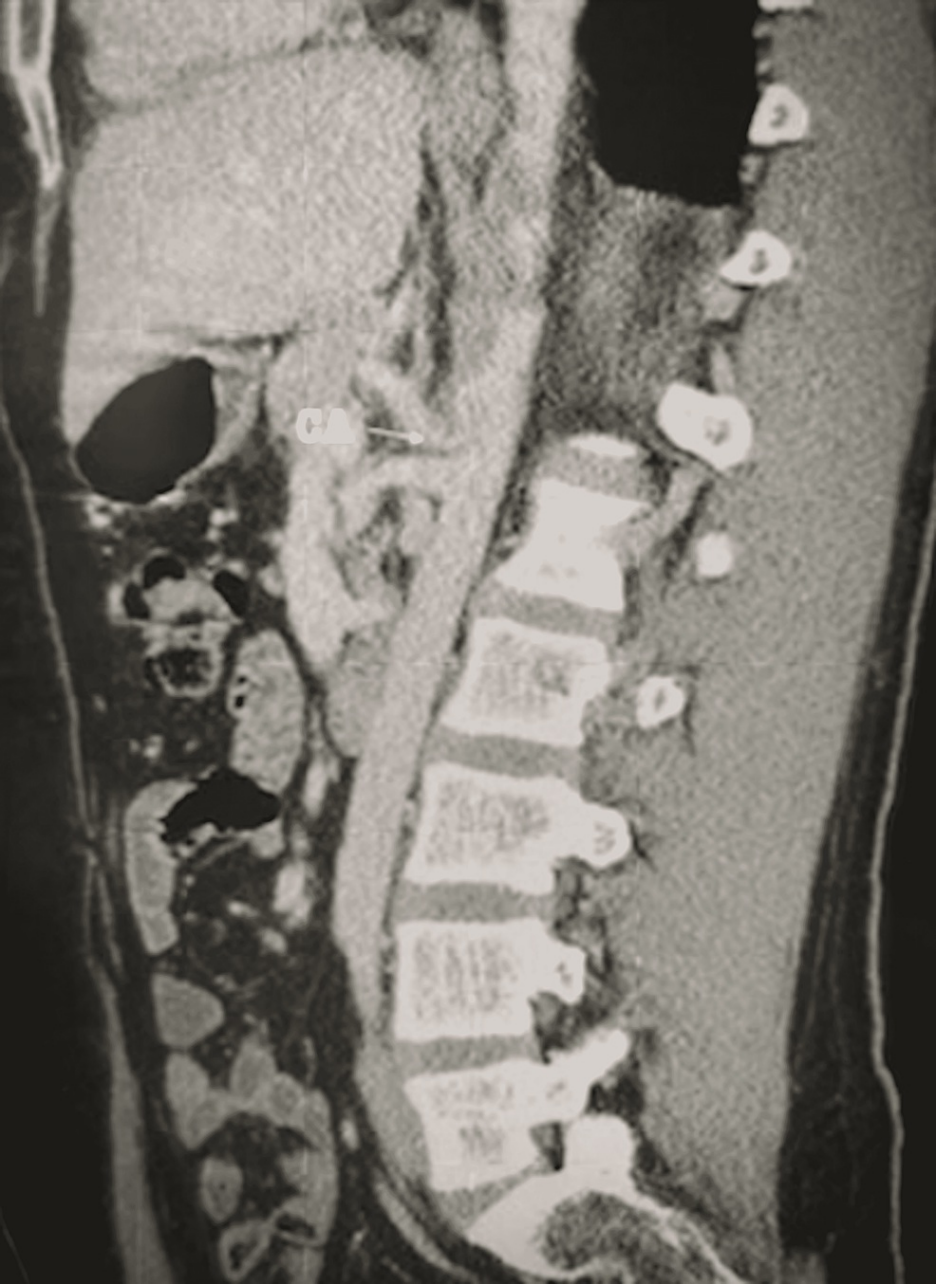
Computed tomography angiography demonstrating 70% stenosis of the celiac artery upon inspiration. CA = celiac artery

The sporadic nature of the episodes and variability of associated symptoms over the course of more than a decade led to a battery of diagnostic workups for multiple system atrophy by various specialists, including multiple neurologists and rheumatologists. However, these diagnostic workups were reported to be negative. Moreover, celiac plexus/ganglion blocks were attempted, and the results were unremarkable. At one point, he was diagnosed with early-onset Parkinson’s disease. Upon evaluation of the patient, the physical exam was unremarkable, but the patient stated that he had lost 20 lbs over the course of the previous few months due to decreased appetite and the development of an aversion to eating. MALS was suspected, and a vascular duplex ultrasound and CT angiography were performed, revealing more than 70% stenosis of the celiac artery and enlargement of the median arcuate ligament, resulting in significant compression of the celiac artery during inspiration (Figure 1).

The decision was made to refer the patient to General Surgery for potential laparoscopic median arcuate ligament release after the patient was determined to be a poor candidate for open surgery. The patient was made aware of the risks and benefits of the procedure, especially due to the proximity of the aorta and the possibility of no symptom relief post-median arcuate ligament release. Consent was obtained and the decision was made to proceed with robotic-assisted celiac artery exposure, median arcuate ligament release, and robotic-assisted celiac neurolysis.

**Figure 2:**
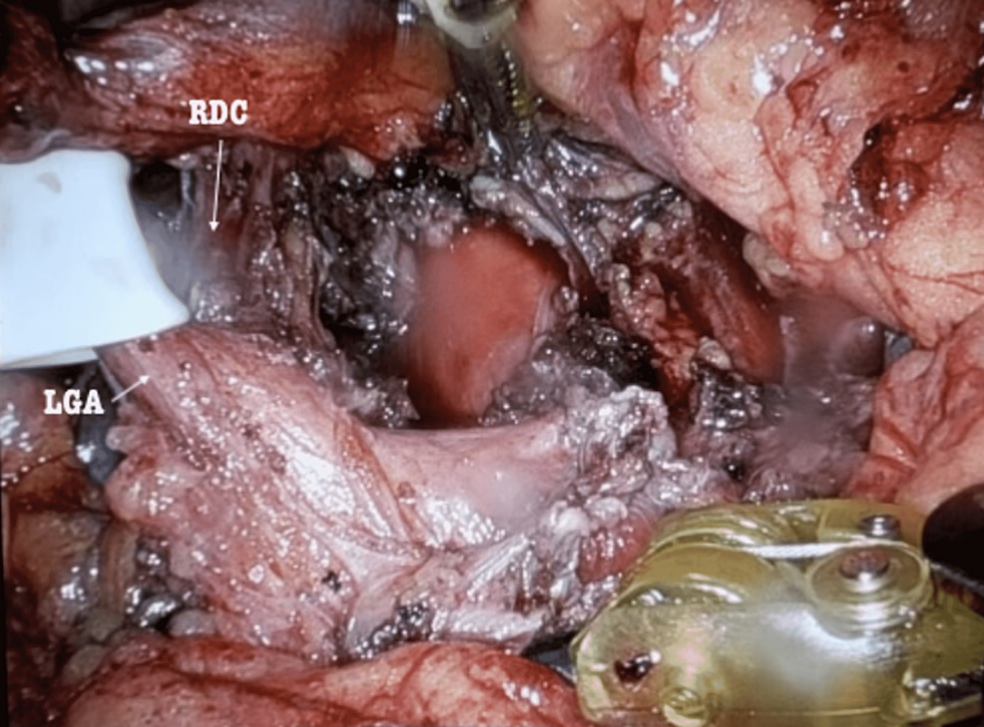
Intraoperative visualization of the fibers of the right diaphragmatic crus. RDC = right diaphragmatic crus, LGA = left gastric artery

The patient was prepped appropriately for surgery and placed in the reverse left Trendelenburg position to allow for appropriate intraoperative exposure. Intraoperatively, the left gastric pedicle was retracted upward to expose the supraceliac area, with direct visualization of the fibers of the right diaphragmatic crus (Figure 2).

**Figure 3:**
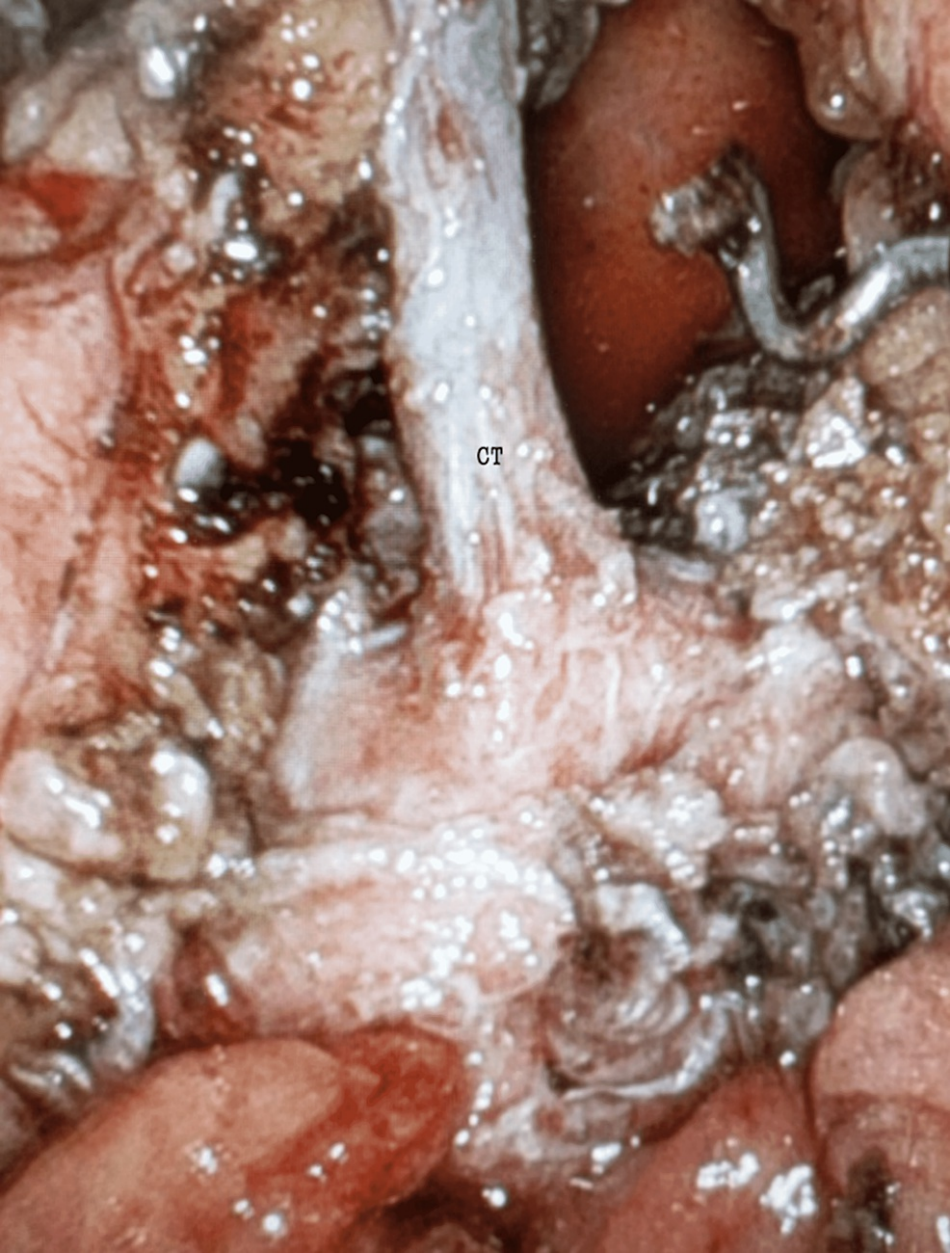
Median arcuate ligament release and celiac neurolysis. CT = celiac trunk

All three branches of the celiac trunk were identified, and attention was directed to the takedown and release of all neuromuscular fibers around the celiac trunk to complete the median arcuate ligament release and celiac neurolysis (Figure 3).

The patient’s postoperative status was stable. He was seen two weeks and two months postoperatively in the clinic. The patient reported complete resolution of his symptoms from as early as his two-week follow-up and confirmed that he had developed no complications and continued to have complete resolution of his symptoms at his two-month follow-up.

## Discussion

As previously indicated, MALS is a rare syndrome with variable and complex etiology and mechanism of disease [7-9]. Of the constellation of symptoms that MALS may present with, postprandial epigastric pain is a classic symptom and increases the index of suspicion for clinicians to engage in a diagnostic workup to assess the compressibility of the celiac trunk in various settings and especially upon inspiration [10-12]. Given that MALS is a diagnosis of exclusion, diagnosis is often delayed or missed altogether, and chronic manifestations of the disease often include progressive weight loss and food aversion [1-3].

In our patient’s case, his constellation of atypical symptoms, including the lack of consistent postprandial epigastric pain and the presence of diffuse, poorly localized abdominal pain and syncopal episodes, in addition to his personal history of ADHD, restless leg syndrome, peripheral neuropathic pain, and family history of epilepsy distracted from MALS as the cause of his presentation and led to a misdiagnosis of Parkinson’s disease. Moreover, the absence of the classic symptom of epigastric pain and the predomination of nonspecific GI symptoms and syncope in his presentation further clouded clinicians’ ability to diagnose MALS. Given that our patient was using gabapentin to treat his peripheral neuropathic pain, and the growing body of evidence supporting a neurogenic component of MALS presentation in some patients, we endeavored to better understand the intersection of our patient’s symptoms and the plausible etiology that resulted in his atypical, chronic MALS presentation [8,9].

Recent evidence presented in a double-blind clinical trial demonstrated the efficacy of gabapentin in reducing the severity of GI symptoms in patients with neurogenic irritable bowel syndrome symptoms, especially pain, reflux, and indigestion [20]. It is, therefore, reasonable to suspect that his use of gabapentin may have served as a symptomatic modifier as its effects on decreasing neurogenic hypersensitivity have been well documented in the literature [20]. This phenomenon may have contributed to the development of poorly localized, diffuse abdominal pain in our patient as neurogenic hypersensitivity in the GI tract has been shown to be mediated through δ2 receptors of calcium channels, which are targets of gabapentin inhibitory drug action [20].

## Conclusions

The atypical and chronic presentation of MALS in our case, in conjunction with our patient’s use of gabapentin, the growing body of evidence presented in the literature supporting the neurogenic etiology of MALS, and recent evidence demonstrating the efficacy of gabapentin in decreasing neurogenic hypersensitivity of the GI tract, represents a novel addition to the literature on the treatment modalities of MALS and elucidates new avenues for continued research. Further research is needed to establish the use of gabapentin as a symptom-modifying agent in the nonoperative and preoperative treatment of MALS, and a reasonable index of suspicion for MALS must be maintained in patients on gabapentin presenting with nonspecific abdominal pain and weight loss.
